# Assessing Auranofin for Second‐Line Use in Chemoresistant Ovarian Cancer: Effects on Tumour Spheroid and Primary Cell Growth

**DOI:** 10.1111/jcmm.70681

**Published:** 2025-06-30

**Authors:** Rosamaria Militello, Matteo Becatti, Tania Gamberi, Tania Fiaschi, Alessandra Modesti, Caterina Paffetti, Flavia Sorbi, Massimiliano Fambrini, Francesca Magherini

**Affiliations:** ^1^ Department of Experimental and Clinical Biomedical Sciences “Mario Serio” University of Florence Florence Italy; ^2^ Obstetrics and Gynecology, Department of Experimental, Clinical and Biomedical Sciences ‘Mario Serio’, University of Florence Careggi University Hospital Florence Italy

**Keywords:** Akt pathway, auranofin, cisplatin chemoresistance, NF‐κB pathway, ovarian cancer, spheroids

## Abstract

Ovarian cancer (OC) is the fifth leading cause of cancer‐related death among women and the most lethal gynaecological malignancy. The high mortality rate is primarily due to late diagnosis and the lack of targeted therapies. The gold standard treatment consists of debulking surgery followed by platinum/taxane‐based chemotherapy, which is initially effective in approximately 75% of patients. However, most women experience relapse and develop chemoresistance. To date, no therapy has proven to be decisive, underscoring the need for research into second‐line or alternative treatments to overcome chemoresistance and prevent relapses. Auranofin (AF) is a promising repositioned anticancer agent with a multifaceted mode of action both cancer cell type‐ and dose‐dependent. The current study evaluated AF's cytotoxicity on multicellular tumour spheroids derived from three ovarian cancer cell lines (SKOV3, A2780, and A2780 cisplatin‐resistant). Results demonstrated that AF inhibited spheroid formation and growth by inducing apoptosis. Furthermore, we showed that AF's mode of action involves the PI3K/Akt and NF‐κB pathways, and we highlighted differences in drug responses between cisplatin‐sensitive, resistant, and primary ovarian cancer cells. Finally, by examining the efficacy of AF and cisplatin in combination, we identified differential sensitivities among the cell lines and primary ovarian cancer cells.

## 1 Introduction

Ovarian cancer (OC) is the fifth leading cause of cancer death in women, making it the most lethal gynaecological malignancy worldwide. This high lethality is primarily due to the advanced stage of the disease at the time of initial diagnosis; in fact, approximately 70% of patients have a disease that has spread beyond the ovaries. The gold standard therapy, consisting of debulking surgery and platinum/taxane‐based chemotherapy, results in initial responses in approximately 75% of patients, but the majority of women experience relapses and succumb to chemoresistant disease. Recently, several studies have described a comprehensive genomic profile of High‐Grade Serous Ovarian Cancer (HGSOC), and patients with Breast Cancer gene (BRCA) mutated or BRCA wild‐type homologous recombination deficient (HRD) tumours have a better prognosis due to the presence of targeted therapy with poly (ADP‐ribose) polymerases inhibitors (PARPis). Despite this, no therapy is currently decisive at the moment, making it imperative that research into ovarian cancer focuses on second‐line or alternative therapeutic strategies capable of overcoming chemoresistance and preventing relapses.

Drug repurposing, also called drug repositioning, is a strategy for identifying new therapeutic applications for approved or investigational drugs, which are different from the original medical indications. This approach permits not only the reduction of time and cost, but above all significantly lowers the risk of failure since the repurposed drug has already been proven sufficiently safe in preclinical models and humans. In fact, for repurposed drugs, detailed information on their pharmacology, formulation, and potential toxicity is already available; thus, preclinical and phase I and II costs and time are substantially reduced, although the regulatory and phase III costs may remain similar to new drugs.

Auranofin (AF), a drug already approved for rheumatoid arthritis, is currently the subject of a broad repositioning plan for cancer therapy [1, 2]. Regarding ovarian cancers, several studies demonstrated AF's ability to overcome cisplatin resistance [3], to block ovarian cancer stem cell phenotypes [4], and to induce redox unbalance, as well as mitochondria and endoplasmic reticulum dysfunctions [5, 6]. Furthermore, our group recently demonstrated that AF induces cell death in A2780 ovarian cancer cell lines and reduces tumour growth in a mouse orthotopic model [7]. Concerning the molecular mechanism of AF, besides the well‐known inhibition of thioredoxin reductase [8], other potential targets, including Inhibitory kB kinase, deubiquitinases, protein kinase C iota, and phosphatidylinositol 3‐kinase/protein kinase B (PI3K/Akt), have been proposed to explain the cytotoxic activity of this drug [1, 2].

Three‐dimensional (3D) in vitro models have been used in cancer research as an intermediate model between in vitro cancer cell lines and in vivo tumours. These models are particularly attractive in ovarian cancer studies due to the unique dissemination pattern of ovarian cancer cells, which rarely involve the vasculature but occur through peritoneal fluid or ascites. Cancer cells, either as single cells or clusters, are shed from the primary tumour into the peritoneal fluid or ascites, where they are passively transported throughout the abdominal cavity. These malignant cells form spheroid‐like structures that settle onto the peritoneal surface, where their disaggregation causes metastatic outgrowth [9]. Spheroids present in ascites are capable of tumorigenesis in vivo and exhibit reduced responsiveness to chemotherapeutic drugs in vitro [10].

Given the peculiar dissemination pattern of OC previously described, in the present study, we evaluated the cytotoxicity of AF on multicellular tumour spheroids of three different ovarian cancer cell lines, SKOV3, A2780, and A2780 cisplatin‐resistant (A2780Cis), as a more realistic model for this kind of tumour. Our study showed that spheroids display increased IC50 values for AF and Cisplatin (Cis) in comparison to two‐dimensional culture, with AF consistently being more effective than Cis. We demonstrated that AF inhibits spheroid formation and growth in a dose‐dependent manner by activating apoptosis. We showed that the PI3K/Akt axis is primarily involved in AF's mechanism of action in two‐dimensional cultures, spheroids, and patient‐derived ovarian cancer cells. Finally, analysing the effect of AF and Cis in combination, we highlighted differences in drug response between A2780 cells sensitive to Cis, their resistant counterpart, and primary ovarian cancer cells.

## 2 Materials and Methods

### 2.1 Cell Lines, Culture Conditions, and Spheroid Generation

Human ovarian cancer cell lines: A2780, the cisplatin resistant counterpart A2780Cis, and SKOV3, were purchased from the European Collection of Authenticated Cell Cultures and were maintained in RPMI‐1640 medium supplemented with 10% of FCS, 2 mM glutamine and antibiotics at 37°C in a 5% CO_2_ atmosphere and sub‐cultured twice weekly. Multicellular tumour spheroids (spheroids) were generated by plating 5000 cells in low attachment plates or by the hanging drop method. For the hanging drop procedure, 25 μL drop of cell suspension with 5000 cells was pipetted onto the lid of the cultivation dish, which is then flipped over onto the dish containing PBS. All cell culture reagents and supplements were purchased from Euroclone.

### 2.2 Tissue Collection and High‐Grade Serous Ovarian Cancer Cell Isolation

Ovarian cancer tissues and ascites were obtained from 6 patients at the time of primary cytoreductive surgery for advanced HGSOC. The patients were enrolled at the Obstetrics and Gynaecology Unit, Careggi University Hospital of Florence. Enrolled patients signed the written informed consent approved by the Tuscany Region Ethics Committee (protocol number 14780).

Tissue samples were collected from areas of clearly macroscopic cancerous tissue. Primary cells were isolated from solid tumours and ascites of different patients. Ascites and cancerous tissues were immediately transferred to the laboratory. Solid samples were maintained in sterile phosphate‐buffered saline (PBS) and processed for cell isolation within 30 min of collection. HGSOC cell isolation from solid tissues (HGSOC_T) and ascites (HGSOC_A) was performed according to the protocol of TG Shepherd et al., with minor modifications [11]. In particular, tissues were digested with dispase II, 2 mg/mL (Sigma, Milan, Italy) in RPMI 1640 medium for 30 min and then cultured in RPMI medium supplemented with 10% of FBS, 2 mM glutamine, and antibiotics.

The medium was changed after 48 h in order to allow ovarian cancer cells to adhere. For cell isolation from ascites, ascitic fluid was mixed 1:1 with complete RPMI 1640 medium. Confluent cells were split 1:2 and always used before passage five.

### 2.3 Image Collection and Analysis

To assess the size of the spheroids, a microscopic analysis was conducted. All image data were collected using a Nikon Eclipse TS100 inverted microscope equipped with a DS‐Fi1 camera (Japan) in TIFF format and analysed with ImageJ2 software version 2.0.0‐rc‐64. At least 20 images per experiment were collected; experiments were performed in triplicate. To distinguish the spheroid area from the background, the function Image‐Adjust‐Colour Threshold was used. The surface of the spheroid was then marked in red. To measure the spheroids' area, the function Analyse Particles was chosen.

### 2.4 Drug Treatments and Cells/Spheroids Viability Assay

Auranofin (AF), Cisplatin (Cis), and all other chemicals were purchased from Merck.

IC50 values were determined by the MTT (3‐(4,5‐dimethylthiazol‐2‐yl)‐2,5‐diphenyltetrazolium bromide) test using an exposure time of 72 h. Briefly, for 2D culture, 10,000 cells were seeded in 96‐well plate, and after 24 h, the medium was replaced with medium containing scalar dilution of drug. For spheroids, 5000 cells were seeded in low‐attachment plate and after 5 days, the medium was carefully replaced with medium containing scalar dilution of drug. Viable cells and spheroids can convert MTT into formazan, which precipitates inside the cells. After 72 h of treatment, the medium was removed and formazan dissolved in DMSO which is detectable at 595 nm. The optical density was read in a microplate reader interfaced with Microplate Manager/PV version 4.0 software (BioRad Laboratories, Hercules, USA). From the absorbance measurements, the half‐maximal inhibitory concentration (IC50) value was calculated using GraphPad Prism software version 6.0 (GraphPad Holdings LLC, USA).

For drug treatments of 2D cultures, 4 × 10^4^/cm^2^ cells were seeded in p100 or in p60 plates, and after 24 h, were exposed to concentrations of AF or Cis for the time indicated in each experiment. Inhibition of spheroid formation and growth was performed by adding AF at the indicated concentrations in the medium used to obtain the drops. In the experiment designed to evaluate the drug effect in pre‐formed spheroids, they were generated in a low‐attachment plate, and after 5 days of growth, treated with AF.

For combination index (CI) determination, 10,000 cells were seeded in 96‐well plates and treated with scalar dilution of AF, Cis, and AF + Cis. CI was then calculated with the method developed by Chou and Talalay [12], using CompuSyn software (www.combosyn.com).

### 2.5 Assessment of Apoptosis and ROS Production by Flow Cytometry

The evaluation of cell death was carried out using the TACS annexin V/propidium iodide staining kit (Trevigen, Gaithersburg, MD, USA) according to the manufacturer's instructions. After treatment, 2D‐cultures were trypsinised, washed with PBS buffer, and suspended in the staining solution for 15 min at room temperature in the dark. Spheroids were directly disaggregated in staining solution. Cells were then washed and immediately analysed by FACSCantoII flow cytometer (BD Biosciences, New Jersey, USA). Cells were gated and plotted on dot plots showing the distribution of annexin V‐positive cells (early apoptotic cells) and annexin V/propidium iodide‐positive cells (late apoptotic cells). Intracellular ROS were measured using 2′,7′‐dichlorodihydrofluorescein diacetate (H_2_DCF‐DA), a cell‐permeable fluorogenic dye able to measure cellular hydroxyl, peroxyl, and other ROS. Within the cell, H_2_DCF‐DA is deacetylated by cellular esterases to a non‐fluorescent compound, which becomes fluorescent once it is oxidised by cellular ROS into 2′,7′‐dichlorofluorescein (DCF).

Cell suspensions from 2D or spheroids (obtained as described for apoptosis assay) were incubated with 5 μM H_2_DCFDA for 30 min in the dark. Then, cells were washed and immediately analysed using a FACSCantoII (BD Biosciences, New Jersey, USA) flow cytometer.

### 2.6 Total Antioxidant Capacity Estimation

The ORAC (oxygen radical absorbance capacity) assay was performed as previously reported [13]. Total antioxidant capacity was calculated using the standard curve based on Trolox concentration.

A microplate Fluorometer (Biotek Synergy H1) was used for fluorescence detection.

### 2.7 Western Blot Analysis

After drug treatments, cells and spheroids were lysed in RIPA buffer supplemented with a cocktail of protease and phosphatase inhibitors. The protein concentrations in the cell lysates were determined by Bradford protein assay kit (Bio‐Rad Laboratories, Hercules, USA) according to the manufacturer's instructions. 4%–20% pre‐cast SDS‐PAGE gels (Bio‐Rad) and PVDF membranes were used for electrophoresis and western blot; PVDF membranes were incubated overnight at 4°C in 2% non‐fat dry milk in PBS‐tween solution containing the following primary antibodies: NF‐κB (1:500, sc‐8008, Santa Cruz), AKT (1:1000, GTX121937‐S, GeneTex), AKT‐phospho‐Ser473‐antibody (1:1000, GTX132615, GeneTex). Membranes were treated with horseradish peroxidase (HRP)‐conjugated secondary antibodies (1:5000 dilution, Santa Cruz) and immunoreactive bands were detected with an ECL kit detection system on an Amersham Imager 600 (GE Healthcare, Chicago, IL, USA). For the quantification, a densitometric analysis of the bands was performed using ImageJ2 software version 2.0.0‐rc‐64 [11]. The intensities of the immunostained bands were normalised on the Coomassie brilliant blue R‐250‐stained total protein from the same PVDF membrane.

### 2.8 Statistical Analysis

GraphPad Prism 6.0 software was utilised to process all the data. Values are reported as mean ± SD of no less than three independent experiments. An independent *t*‐test was employed to compare the two groups, whereas one‐way analysis of variance (ANOVA), followed by Tukey's multiple comparisons, was used to compare data among multiple groups. A *p*‐value ≤ 0.05 was considered statistically significant. For details, see the figure legends.

## 3 Results

### 3.1 AF Induces Viability Reduction in Ovarian Cancer Cell Lines and Spheroids

**FIGURE 1:**
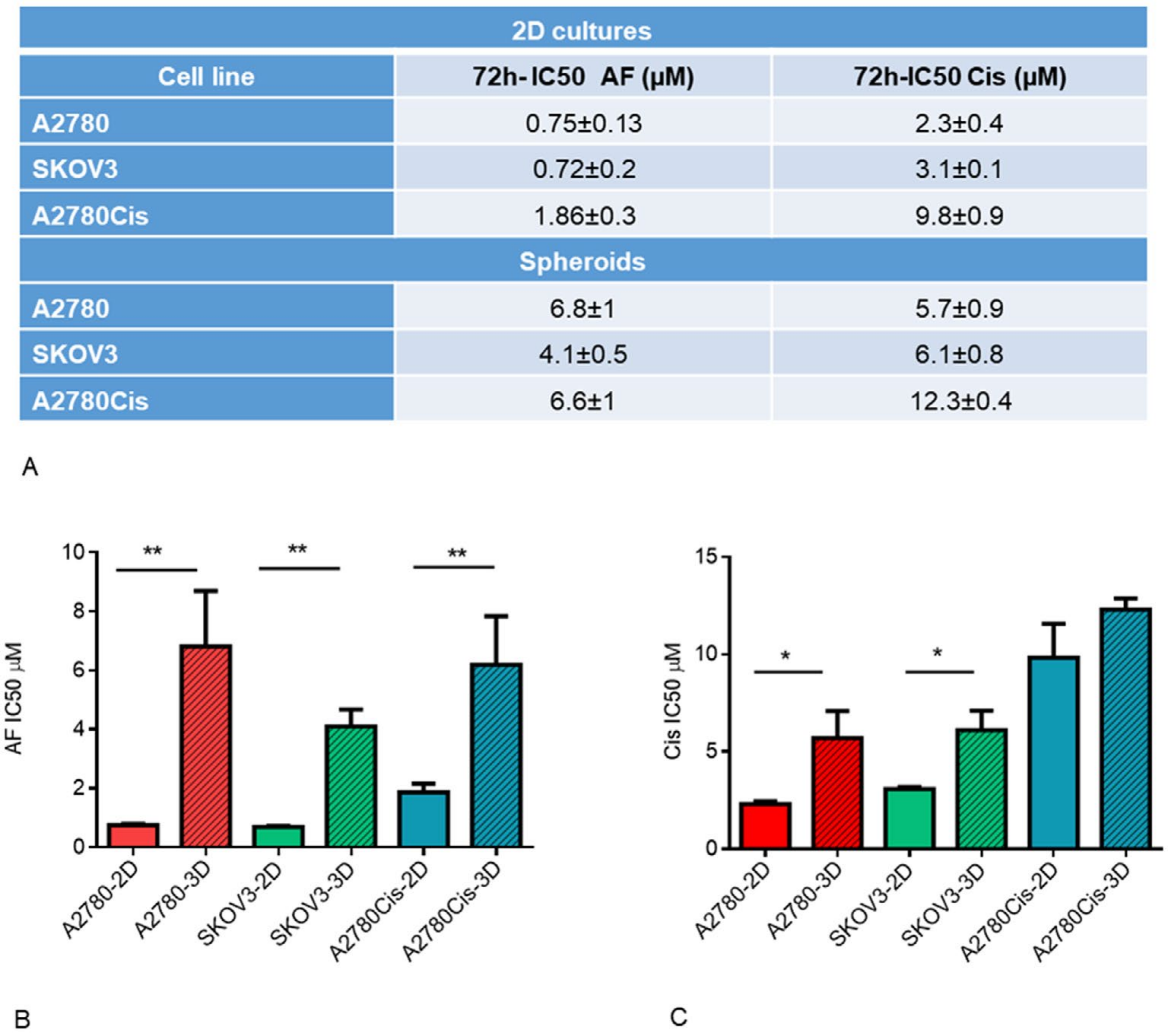
2D cultures and 3D cultures (spheroids) show different sensitivity to AF and Cis. (A) IC50 values of 2D culture and spheroids after 72 h of treatment with AF or Cis by MTT test. (B, C) Histograms represent the difference between IC50 values of 2D and spheroids treated with AF and Cis, respectively. Means and SD are reported; GraphPad Prism software was used for IC50 determination and statistical analysis. *p < 0.05, **p < 0.01.

The cytotoxicity of AF was evaluated on three ovarian cancer cell lines in two‐dimensional (2D) and three‐dimensional (3D, spheroids) conditions by MTT assay. For comparison purposes, the cytotoxicity of Cis was also evaluated. In particular, cells were treated with different drug concentrations, and the IC50 after 72 h was determined (72 h‐IC50). The IC50 values are reported in Figure 1A, and representative dose–response curves are shown in Figure S1.

In A2780 and SKOV3 2D cultures, the concentration of AF required to reduce cell viability by 50% was more than three times lower than that of Cis. The A2780Cis cell line exhibited a 2‐fold increase in AF's IC50, whereas Cis' IC50 is about 5‐fold higher, suggesting that AF could overcome Cis resistance. However, the induction of Cis resistance is accompanied by a slight increase in resistance to AF as well.

In spheroids, the AF IC50 values strongly increased in comparison to 2D cultures (a 6‐fold and a 9‐fold increase for SKOV3 and A2780, respectively). The IC50 values of spheroids derived from A2780Cis increased 3‐fold (Figure 1B). Regarding Cis, the IC50 of spheroids presented a less pronounced increase compared to AF, which was not significant for the resistant cell line (Figure 1C).

### 3.2 AF Reduces Tumour Spheroid Growth

**FIGURE 2:**
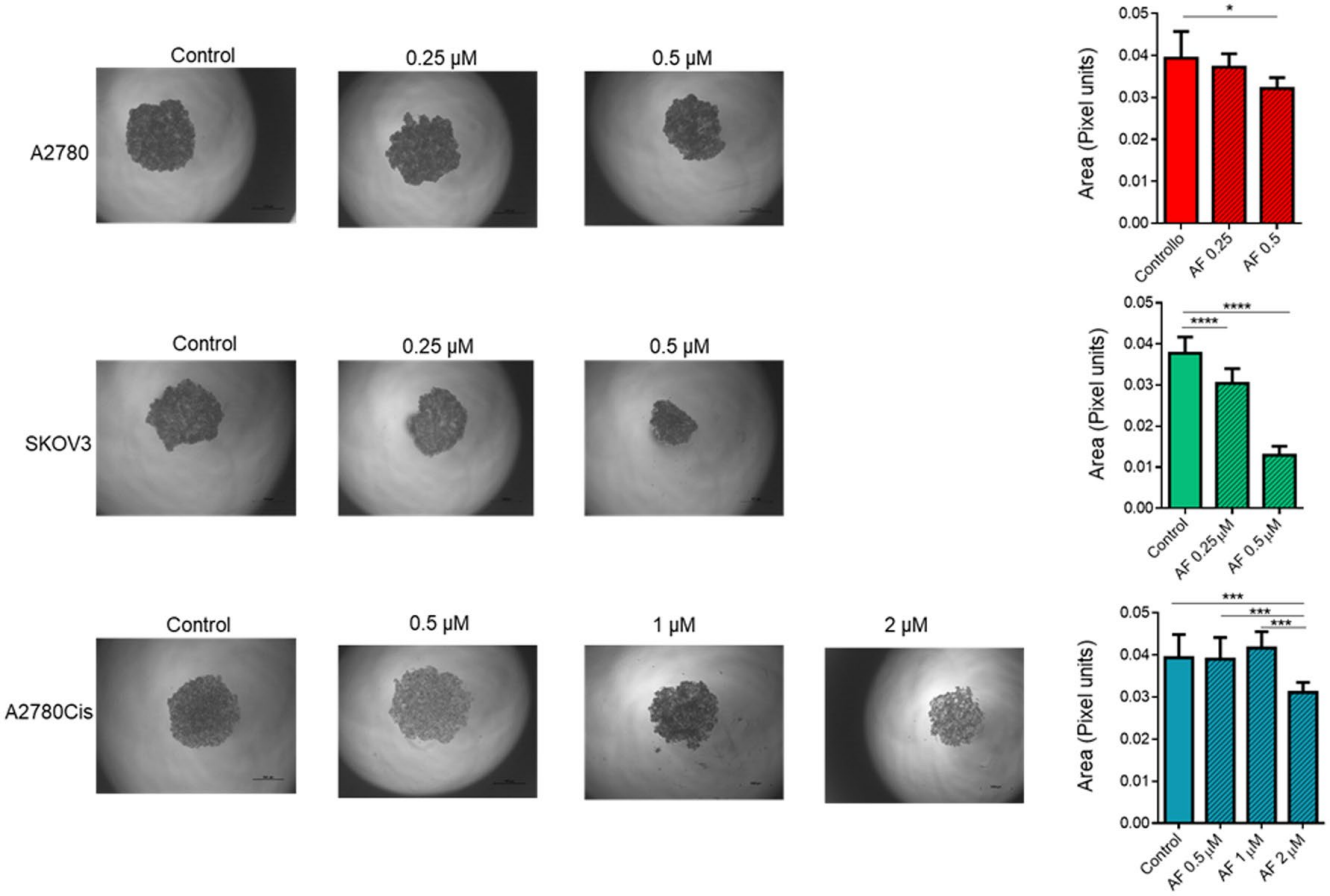
AF affects spheroid growth. Spheroids were obtained by the hanging drop method starting from 5000 cells. Spheroid growth was performed in medium containing increasing drug concentrations. (A) Representative microscope images (4×). (B) Mean values of spheroid, area ± SD, was obtained evaluating spheroid area of at least 20 spheroids in three independent experiments. Scale bars represent a length of 1 mm. *p < 0.05, **p < 0.01, ***p < 0.001.

In ovarian cancer, spheroids found in ascites may derive from the aggregation of cancer cells; thus, in order to evaluate whether AF can impede cell aggregation, the hanging drop method was used to induce spheroid formation in culture media containing AF. The comparisons among formed spheroids were made by measuring spheroid area (in pixels) after 96 h of growth (Figure 2). A2780 spheroids showed a statistically significant area reduction of 18% with 0.5 μM AF compared to control. SKOV3 spheroids exhibited significant reductions of 20% and 65% with 0.25 and 0.5 μM AF, respectively. As expected, A2780Cis spheroids were less sensitive, showing an area reduction of 25% when treated with 2 μM AF.

### 3.3 The Inhibition of Spheroid Growth due to AF Is Associated With Apoptotic Cell Death and ROS Production

**FIGURE 3:**
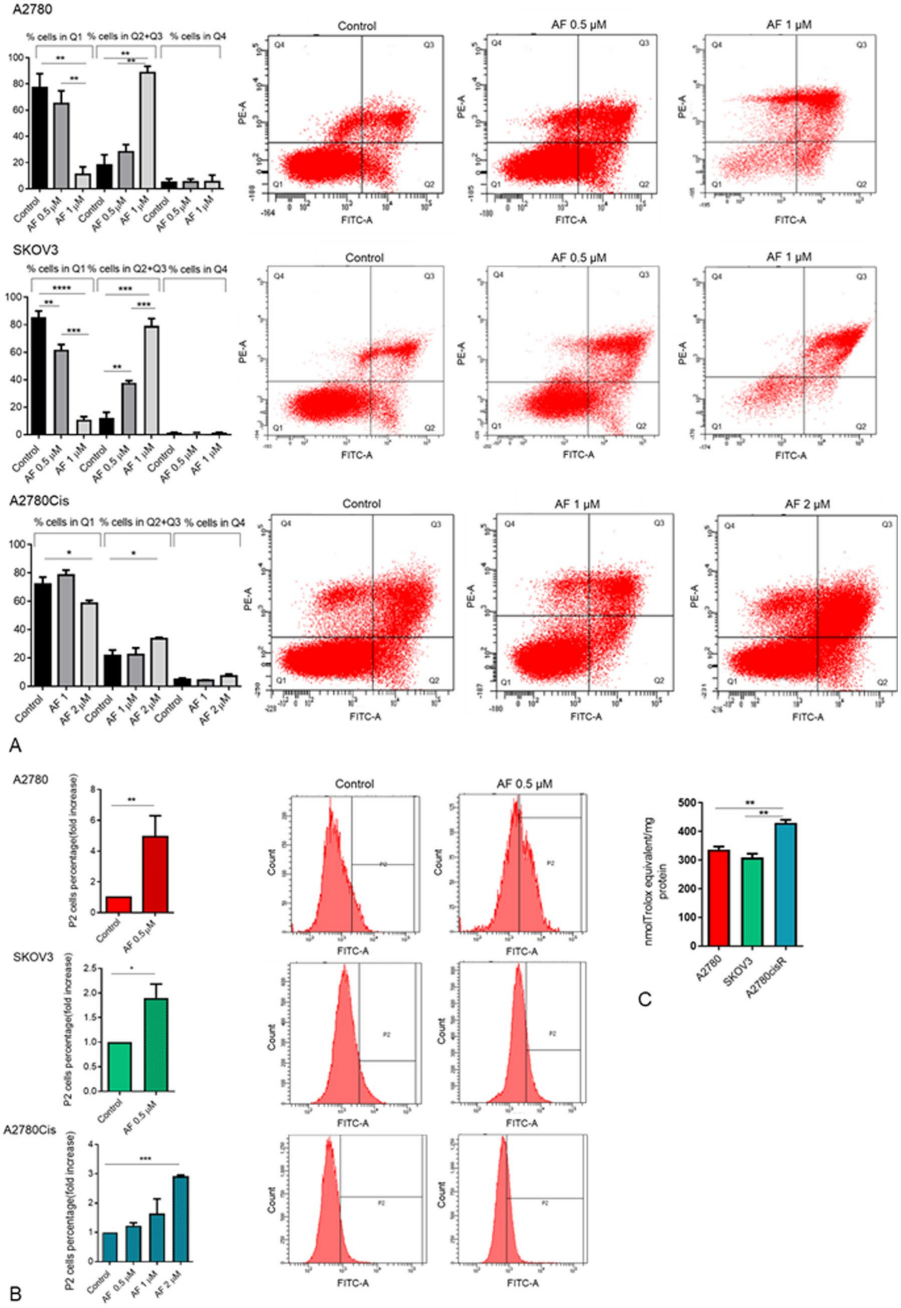
AF impairs spheroid formation and growth by inducing apoptotic cell death and ROS production. (A) Percentage of apoptotic and necrotic cells shown by flow cytometry analysis of annexin V/propidium iodide‐stained. Spheroids were grown for 72 h with increasing AF concentrations. Flow cytometric images are representative of three independent experiments. Histograms report the mean values ± SD. (B) ROS production during spheroid formation and growth was evaluated using the DCFDA probe. Histograms represent the percentage of P2 (ROS‐positive) cells. (C) Total antioxidant capacity (ORAC assay). Histogram reports the mean values ± SD of three independent experiments. *p < 0.05, **p < 0.01, ***p < 0.001.

To verify whether spheroid growth reduction was due to the arrest of cell proliferation or to apoptosis, the spheroids were grown for 72 h with increasing AF concentration. They were then harvested, gently dissociated with a pipette, and stained with annexin V and propidium iodide to analyse apoptosis. As shown in Figure 3, for A2780 and SKOV3 cells, spheroid formation in the presence of 1 μM AF resulted in a percentage of cells in early and late apoptosis (Q2 + Q3) of 6‐fold (SKOV3) and 5‐fold (A2780) higher than in the control, whilst 0.5 μM AF induced a significant increase of cell death only in SKOV3 cells. A2780Cis spheroids were more resistant to AF treatment, but AF 2 μM induced a 2‐fold increase in apoptotic cells in comparison to the control. On the other hand, when A2780Cis spheroids were treated with 2 and 3 μM of Cis, no increase in apoptotic cells was detected (Figure S2). Several studies have reported that AF treatment generates an increase in ROS production that could contribute to the activation of the apoptotic process. To verify if AF could induce ROS production during spheroid formation, the ROS amount was evaluated using the DCFDA probe in spheroids. As shown in Figure 3B, AF induced an increase in ROS production in spheroids from all tested cell lines. In accordance with the apoptosis results, the concentration of AF necessary to induce ROS production in A2780Cis spheroids was higher than that used for the non‐resistant cell lines. These results suggested that A2780Cis cells could have developed a more antioxidant environment. To confirm this hypothesis, the total antioxidant capacity was tested with the ORAC assay. As shown in Figure 3C, A2780Cis cell line has a higher total antioxidant capacity than A2780 and SKOV3 cell lines.

### 3.4 AF Induces Apoptotic Cell Death in Pre‐Formed Tumour Spheroids

**FIGURE 4:**
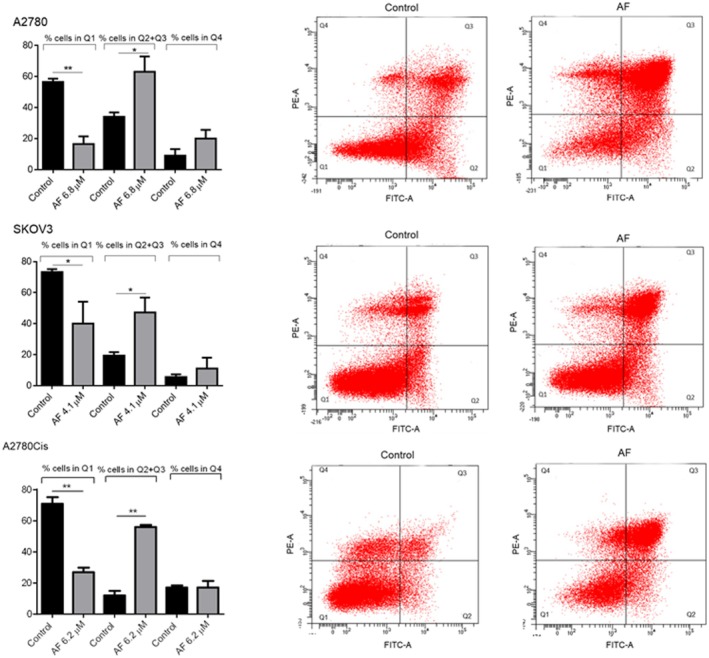
AF induces necrotic and apoptotic cell death in pre‐formed spheroids. Percentage of apoptotic and necrotic cells shown by flow cytometry analysis of annexin V/propidium iodide‐stained. Spheroids were treated for 72 h with AF concentrations corresponding to IC50 values. Flow cytometric images are representative of three independent experiments. Histograms report the mean values ± SD. *p < 0.05, **p < 0.01.

In section 3.2, we demonstrated that AF reduces spheroid formation; as a second step, we verified the ability of AF to induce cell death in pre‐formed spheroids. For this purpose, spheroids were formed starting from 5000 cells in low attachment 96 multiwall plates and grown for 5 days before AF treatment was used at the 72 h‐IC50 concentration specific for spheroids of each cell line. Apoptosis was detected 72 h later. Results are reported in Figure 4. In addition to apoptotic cells, the presence of necrotic cells is also detectable; this is a well‐described feature of spheroids in which the innermost cells, being excluded from the supply of nutrients and gaseous exchanges, undergo necrotic death [14]. Despite this, the effect of AF is clearly detectable with a reduction of viable cells of 71% for A2780, 45% for SKOV3 and 68% for A2780Cis.

### 3.5 AF Affects Akt and NF‐κB Pathways

**FIGURE 5:**
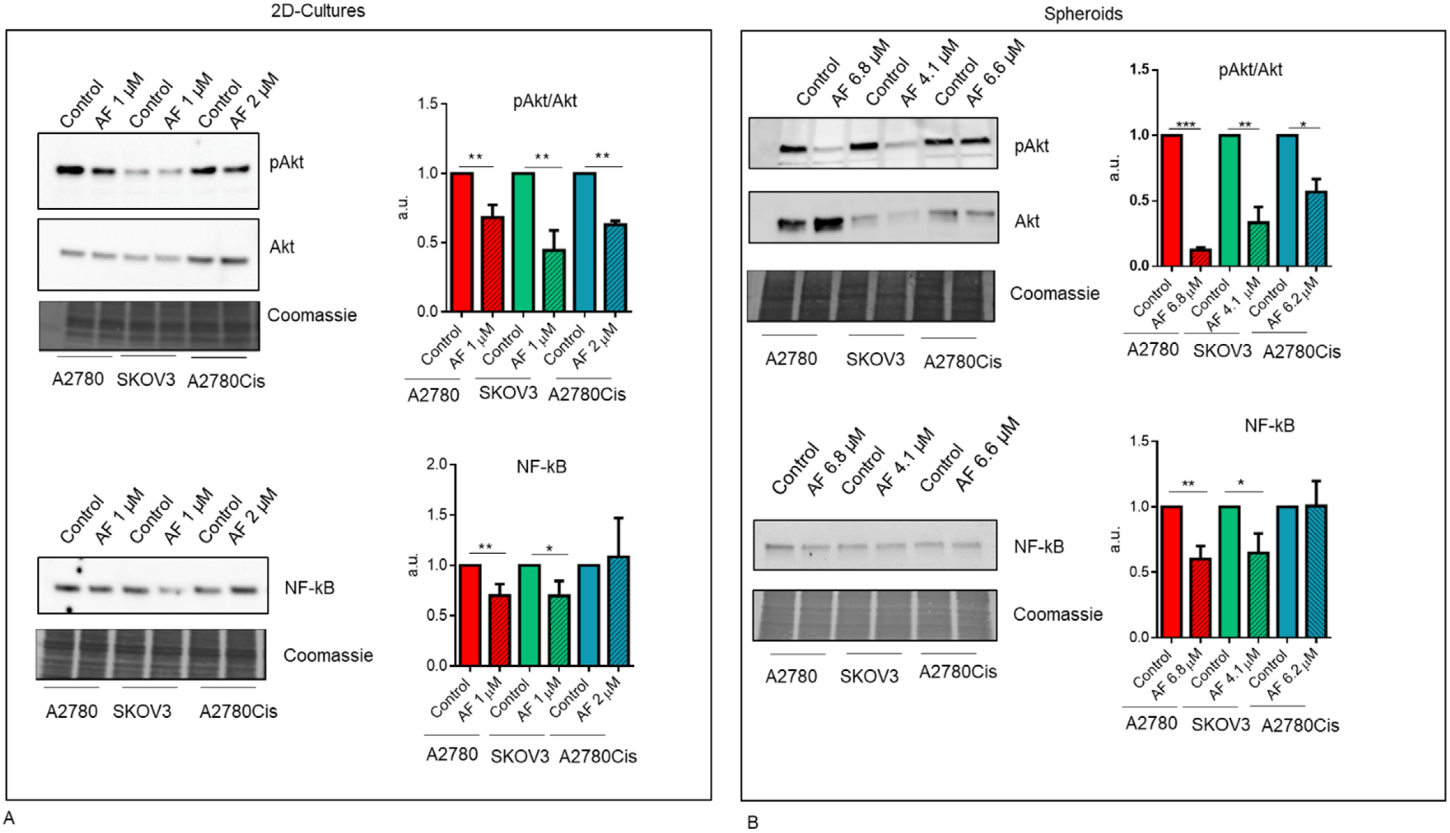
AF impairs Akt signalling both in 2D culture and in spheroids. Protein level of phosphorylated Akt (pAkt), Akt, and NF‐κB was evaluated by Western blot analysis in 2D cultures (A) and in spheroids (B). Images are representative of three independent experiments. Histograms report the mean ± SD of at least three independent experiments. *p < 0.05, **p < 0.01, ***p < 0.001.

Although ovarian cancer is a heterogeneous neoplasm with distinct molecular features, several studies suggest that in HGSOC, pathways involving NF‐κB and the PI3K/AKT axis play a pivotal role in cancer progression [15, 16]. Since AF has been proven to affect both these signalling pathways, albeit in different cell contexts [1], we investigated whether the induction of cell death by AF could be linked to their impairment. To achieve this aim, we treated for 24 h cells grown in a monolayer and spheroids with AF at the indicated concentrations. As shown in Figure 5A, cells grown in 2D conditions demonstrated a decrease in phosphorylated Akt level upon AF treatment, and in agreement with apoptosis data, in A2780Cis, the decrease is observed with AF 2 μM (AF 1 μM has no significant effect, data not shown). NF‐κB level is not affected in A2780Cis treated with the indicated AF concentrations.

### 3.6 Ovarian Cancer Cells From Patients Display the Same Features of Ovarian Cell Lines

Since established cell lines may not fully reflect the behaviour of cancer cells, we validated some of the main findings on epithelial ovarian cancer cells (EOC) isolated from patients. EOCs were isolated from both ascites and tumour tissues of 6 different patients with HGSOC. Cells were maintained in culture for a maximum of five passages. We first evaluated the IC50 of 2D cultures in order to figurate whether, regarding cisplatin sensitivity, they were more similar to sensitive or resistant cell lines. As reported in Figure 5A, concerning 72 h‐IC50, the primary EOC analysed resulted more similar to the A2780Cis cell line.

**FIGURE 6:**
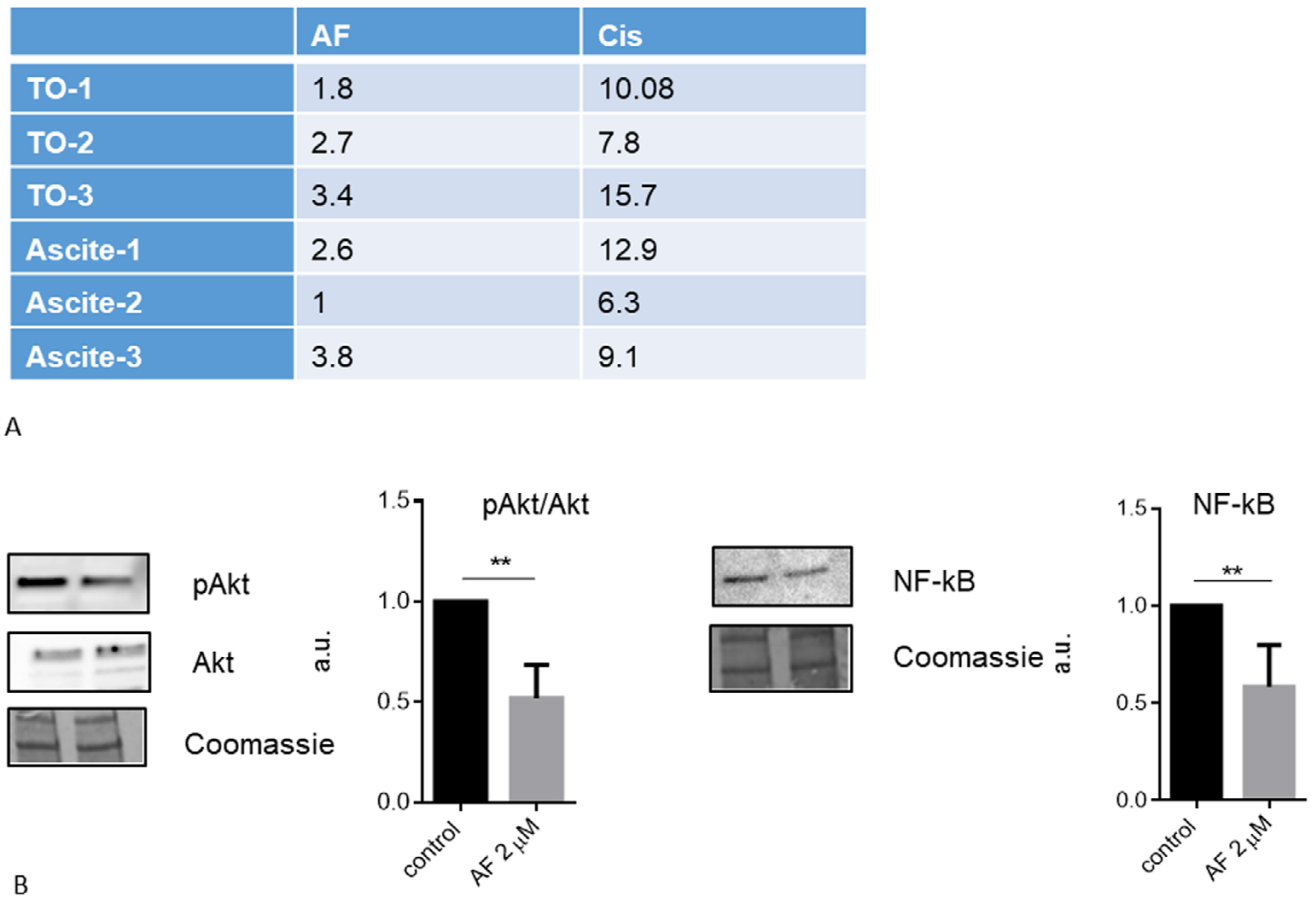
AF impairs Akt and NF‐κB signalling in EOC cells. (A) 72 h‐IC50 values of primary epithelial ovarian cancer cells isolated from solid tumour (TO) or ascites. (B) Protein level of phosphorylated Akt (pAkt), Akt, and NF‐κB was evaluated by western blot analysis in EOC cells. Histograms report the mean values ± SD of cells derived from three different patients. *p < 0.05, **p < 0.01.

Concerning AF impact on Akt phosphorylation and NF‐κb signalling, both pathways are impaired by AF, supporting the potential translation of these results in clinical practice (Figures 5B and 6).

### 3.7 The Effects of Combining AF and Cis Depend on Cell Types

**FIGURE 7:**
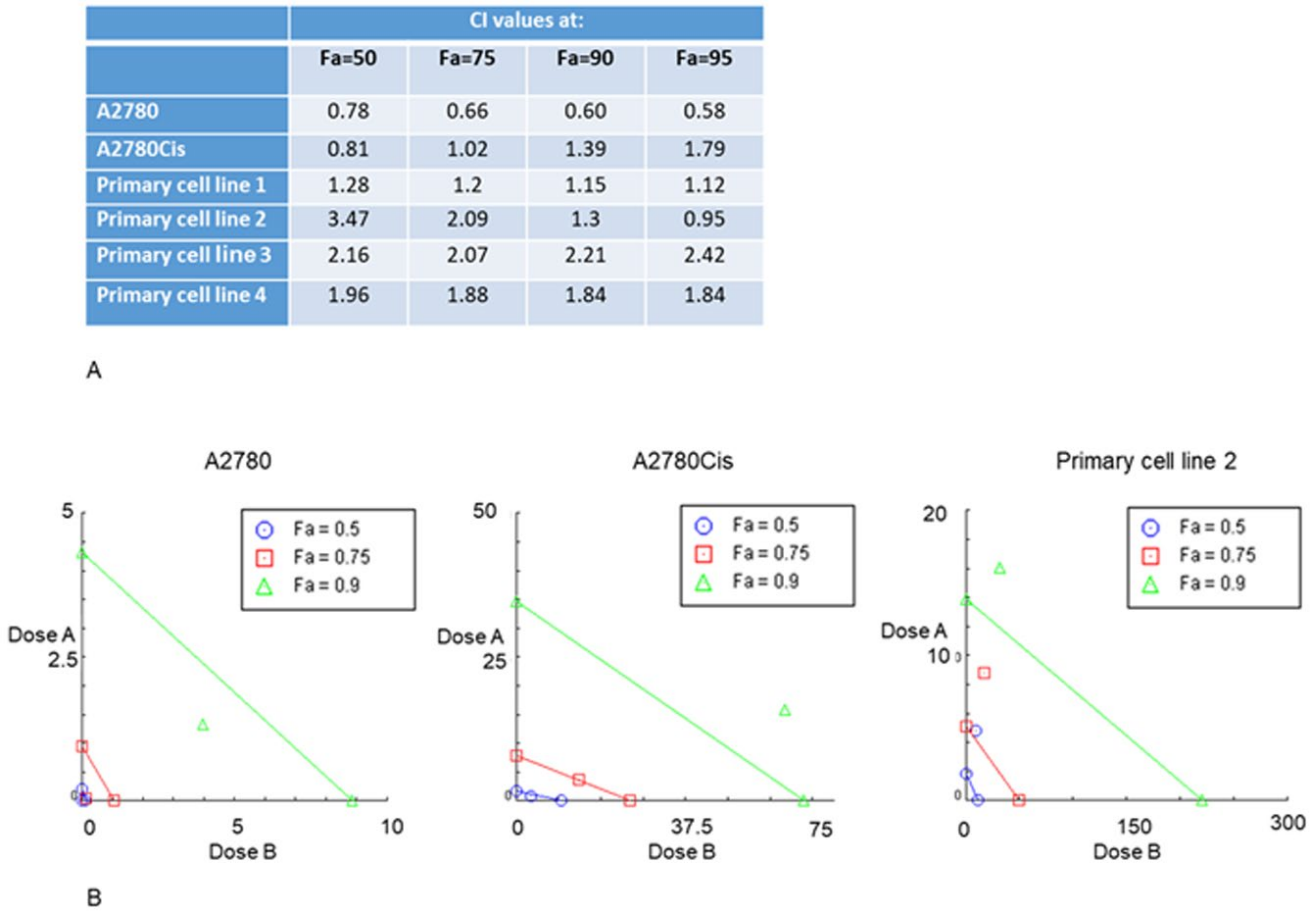
AF and Cis combination results additive/antagonistic in A2780Cis and in primary cell lines. (A) CI values for drug combinations that induce a fraction inhibition (Fa) of 50%, 75%, 90%, and 95% evaluated by MTT assay. (B) Examples of isobolograms for Fa of 50%, 75%, and 90%. Combination data points on the upper right of the diagonal indicate antagonism, whilst data points on the lower left indicate synergism (more details are present in the text).

In the context of drug repurposing, it is also important to evaluate whether the drug object of the study can potentially be used in combination with the standard chemotherapy treatment. Thus, CompuSyn (ComboSyn Inc., Paramus, NJ, USA) method was used to identify whether AF either acts synergistically (CI < 1), additively (CI = 1), or antagonistically (CI > 1) with cisplatin [12]. As shown in the previous section, primary cells are more similar to A2780Cis cell line than to A2780 in terms of IC50 values. Thus, we performed a CI calculation on A2780, A2780Cis, and 4 primary ovarian cancer cells isolated from patients. As reported in Figure 7, AF and Cis appear synergic on A2780 cells with CI always below 1, but appear additive and/or antagonistic in A2780Cis and also in primary ovarian cancer cells.

## 4 Discussion

OC has a unique process of metastasising. Although a new model of hematogenous ovarian cancer spread has been recently demonstrated by Coffman et al., OC metastasises preferentially via intraperitoneal dissemination and is only superficially invasive [17, 18]. Single tumour cells or clusters detached from the primary tumour are transported passively through the physiological movement of peritoneal fluid and disseminate to the peritoneum and omentum [9]. These clusters form spheroid‐like structures and have a pivotal role in cancer dissemination and chemoresistance. Several studies have shown that spheroids harbour an increased resistance to paclitaxel and cisplatin, which are currently used in ovarian cancer therapy [19, 20, 21]. Given these particular features of OC, it is crucial to evaluate the effect of potential new drugs on spheroids to gain a more comprehensive understanding of the drug's efficacy and mechanism of action.

AF is now the subject of numerous studies aimed at its repositioning. It is well‐known that the main target of AF is Thioredoxin reductase (TrxR), but besides this enzyme, other targets/pathways have been proposed, suggesting a more complex overall action of this drug [1, 22]. We previously reported that the specific genetic background of cell types could be important in determining the primary AF targets [1]. In particular, using data from the Connectivity Map (CMAP) website (http://clue.io/cmap), we showed that the transcriptional expression profile of A549 lung cancer cells exposed to AF displays effects very similar to TrxR silencing, whereas MCF7 breast cancer cells show a very low correlation. Furthermore, transcriptomic profiles of 45 different cancer cell lines treated with AF generate three different clusters of transcriptomic profiles, one of which includes proteins involved in proliferative pathways mediated by the PI3K/Akt axis. Alia Ghoneum and Neveen Said have recently reviewed the implication of the PI3K/Akt/mTOR pathway and their interconnection with NFκB signalling in OC. In their study, they examined the OC gene mutation profile generated from datasets in cBioPortal (http://www.cbioportal.org) and showed that several proteins in the PI3K/Akt/mTOR/NF‐κB pathway were hyperactivated [15]. Interestingly, both NF‐κB and Akt have been suggested as possible players in the mechanism and anticancer activity of AF. In particular, Joen et al. demonstrated that AF binds Cys1789 of IkB kinase, blocking its activity and the related activation of NF‐κB [23]. Following this publication, several articles pointed out a reduction in NF‐κB protein levels after AF treatment in cancers such as multiple myeloma and lymphoma [24, 25]. Additionally, in lung cancer, breast, and hepatoma, AF was shown to reduce the phosphorylation level of Akt [26, 27, 28, 29]. Recently, Su Z et al. identified critical cysteine residues of Akt that are essential for its plasma membrane recruitment and activation; if these residues were oxidised upon AF‐induced oxidative stress, its recruitment and activation was impaired [30].

As recently reported by Rinne N et al., amplification of the *AKT2* gene was identified in 18.2% of patients with HGSOC [16]. Survival analysis of advanced stage HGSOC (stages 3 + 4) in TCGA data using Kaplan–Meier (KM) plot (http://kmplot.com/analysis) indicates that higher expression levels of Akt isoforms are associated with lower patient survival rates [15], datum which could be related to Akt's involvement in OC chemoresistance [31]. Overall, these findings suggest that Akt and NF‐κB could be therapeutic targets for ovarian cancer.

In this context, our study demonstrated that AF, at a dose comparable to those achieved with standard treatment for rheumatoid arthritis, induces apoptosis during spheroid formation and in pre‐formed spheroids, indicating that this drug could be effective against these structures, which play an important role in OC metastasis. Here we show that two potential targets in ovarian cancer therapy, Akt and NF‐κB, are impaired after AF treatment. In particular, the impairment of pAkt and NF‐κB proteins by AF was detected not only in ovarian cancer cell lines but also in spheroids and, most importantly, in ovarian cancer cells isolated from HGSOC patients.

Considering the possibility of combined treatment with drugs currently used in OC therapy, our results suggest that the combination with cisplatin could be counterproductive. In fact, we used the Chou‐Talalay algorithm for drug combination, based on the median‐effect equation, to predict if the effect of AF and Cis on ovarian cancer cells is additive, synergic, or antagonistic [12]. This method provides the theoretical basis for the combination index (CI) determination of drug interactions, where CI < 1, = 1, and > 1 indicate synergism, additive effect, and antagonism, respectively. In our study, the combination resulted synergic in A2780 but additive and/or antagonistic in A2780Cis and in primary ovarian cancer cells. Interestingly, primary cells were more similar to A2780Cis, suggesting that the resistant counterpart represents a more realistic model of ovarian cancer, as we suggested in our recent publication on the metabolomic profile of OC primary cell cultures and A2780 sensitive and resistant to Cis [32]. The present study also demonstrates that AF can overcome Cis resistance, although not completely. In fact, A2780Cis cells show an increased AF IC50 compared to A2780, indicating that Cis resistance also partially affects AF resistance. Nevertheless, the concentration of AF able to induce cell death in A2780Cis and in primary ovarian cancer cells ranges between 1 and 3.8 μM, a concentration consistent with the plasma and faeces concentrations of AF achieved during rheumatoid arthritis therapy, thus suggesting potential clinical application [33]. To date, a search on www.clinicaltrial.gov reveals five studies repurposing AF in cancer therapy, two of which are in OC. In a completed pilot study on 10 patients (NCT01747798) in which AF was used alone, one patient showed a clear reduction of CA125 [34]; another ongoing study (NCT03456700) is using AF in combination with the immunosuppressive drug Sirolimus. Overall, our results on AF and Cis combination discourage their combined use, suggesting alternative solutions for potential in vivo translation of our results. Interesting possibilities include using AF in second‐line therapy when the tumour has developed platinum resistance or stratifying patients to identify those most likely to respond to AF, either alone or in combination with a non‐platinum drug.

Although further investigation is needed to translate preclinical data into patient treatments, our data suggest that it is worth exploring the possibility of repositioning AF in OC therapy.

## Author Contributions

**Rosamaria Militello:** data curation (supporting), investigation (supporting), methodology (equal), writing – review and editing (equal). **Matteo Becatti:** data curation (equal), methodology (equal), writing – review and editing (equal). **Tania Gamberi:** conceptualization (supporting), funding acquisition (supporting), methodology (equal), writing – review and editing (supporting). **Tania Fiaschi:** methodology (equal), writing – review and editing (supporting). **Alessandra Modesti:** funding acquisition (supporting), writing – review and editing (equal). **Caterina Paffetti:** investigation (equal), methodology (equal). **Flavia Sorbi:** methodology (equal), resources (supporting), writing – review and editing (supporting). **Massimiliano Fambrini:** resources (equal). **Francesca Magherini:** conceptualization (lead), funding acquisition (equal), supervision (lead), writing – original draft (lead).

## Conflicts of Interest

The authors declare no conflicts of interest.

## Supporting information


**Figure S1.** Representative IC50 dose–response curves.

**Figure S2.** Cisplatin, used at the indicated concentrations, failed to induce spheroid cell death. Percentages of apoptotic and necrotic cells, shown by flow cytometry analysis of annexin V/propidium iodide‐staining. Spheroids were grown for 72 h with increasing concentrations of Cisplatin. Flow cytometric images are representative of two independent experiments. Histograms report the mean values ± SD.

## Data Availability

The authors have nothing to report.
