# Supplementary figures and images for: Assessing Auranofin for Second‐Line Use in Chemoresistant Ovarian Cancer: Effects on Tumour Spheroid and Primary Cell Growth
Source: J Cell Mol Med. 2025 Jun 30;29(13):e70681. doi: 10.1111/jcmm.70681 (PMC12207987; doi:10.1111/jcmm.70681)

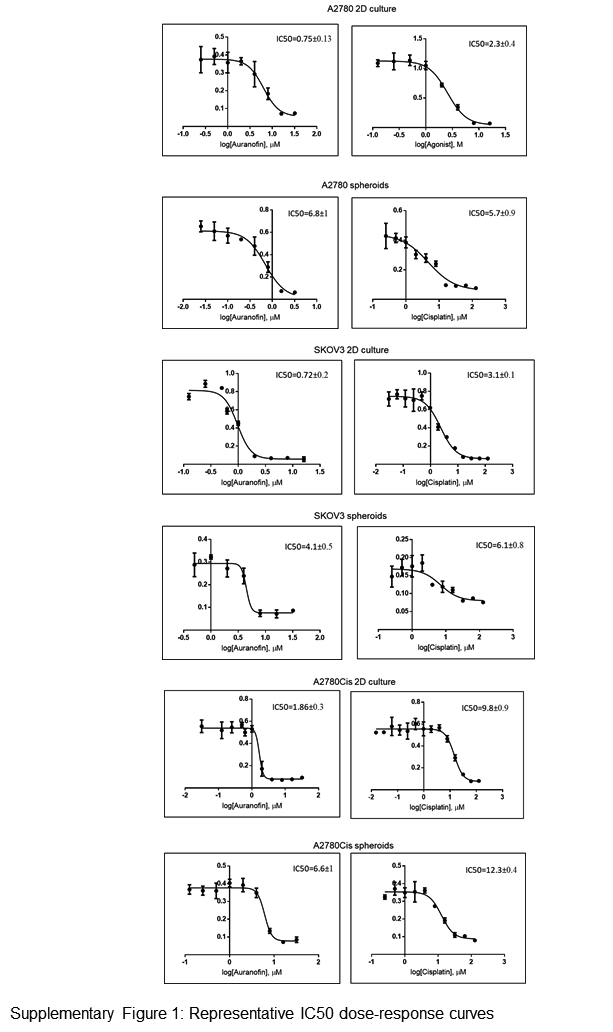

Supplement: Supplementary file 1 — Figure S1. Representative IC50 dose–response curves. [file JCMM-29-e70681-s002.tif]

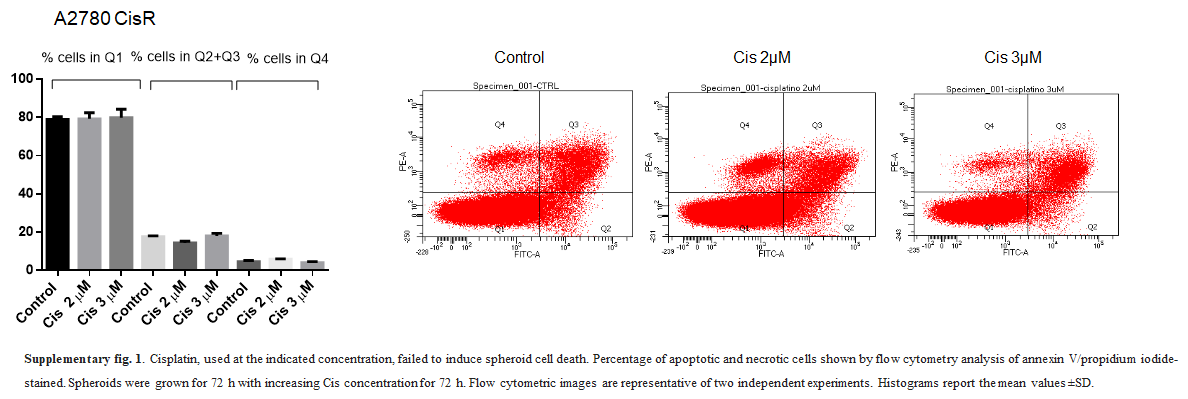

Supplement: Supplementary file 2 — Figure S2. Cisplatin, used at the indicated concentrations, failed to induce spheroid cell death. Percentages of apoptotic and necrotic cells, shown by flow cytometry analysis of annexin V/propidium iodide‐staining. Spheroids were grown for 72 h with increasing concentrations of Cisplatin. Flow cytometric images are representative of two independent experiments. Histograms report the mean values ± SD. [file JCMM-29-e70681-s001.tif]
